# Cognitive impairments associated with chemotherapy in women with breast cancer: a meta-analysis and meta-regression

**DOI:** 10.1590/1414-431X2023e12947

**Published:** 2023-10-13

**Authors:** M.E.C. Oliveira, G.S.V. Torres, R.G. Franklin, K.A.L. Gomes, W.F.S. Nóbrega, T.P. Fernandes, N.A. Santos

**Affiliations:** 1 Departamento de Psicologia, Universidade Federal da Paraíba, João Pessoa, PB, Brasil; 2 Departamento de Psicologia, Universidade Federal de Pernambuco, Recife, PE, Brasil; 3 Departamento de Odontologia, Universidade Estadual da Paraíba, Campina Grande, PB, Brasil

**Keywords:** Breast cancer, Chemotherapy-related cognitive impairment, Cognition, Mental health, Neuroscience

## Abstract

Chemotherapy is one of the most widely used treatments for breast cancer (BC). However, there is evidence of side effects like cognitive changes related to the chemotherapy treatment. The aim of the study was not only to summarize the existing evidence on the relationship between chemotherapy and cognitive performance in women with BC but also to identify additional consequences and aspects associated with these impairments. We conducted a systematic review with meta-analysis and meta-regression to present updated information on the matter. We retrieved data from the databases PubMed, Web of Science, PsycINFO, CINAHL, and Scopus. Twenty studies comprising over 2,500 women were examined and the results indicated that chemotherapy can compromise cognition in women with BC (-1.10 OR [95%CI: -1.81 to -0.74], P<0.01), with working memory (-0.49 OR [95%CI: -0.85 to -0.13], P=0.03) being the most affected among the domains. Furthermore, additional data indicated that cognitive impairment is most likely amid women with BC having a lower education level (Q=4.85, P=0.02). Our results suggested that chemotherapy affects cognitive functions in women with BC, and certain characteristics can worsen the deterioration. A comprehensive study of women with breast cancer and existing predictors contributes to optimized personal journeys, elevated life prospects, and advanced care that can also aid prognosis and therapeutic approaches.

## Introduction

Breast cancer (BC) is characterized by unrestrained cellular proliferation and infiltration of abnormal cells into surrounding tissues and organs (1). It is the most prevalent type of cancer in the female population. Notably, it surpassed lung cancer as the most commonly diagnosed cancer in 2020, with a staggering 2.3 million new cases annually (2). Chemotherapy is considered the gold standard therapy approach for BC, targeting to suppress abnormal cell growth (3). However, its systemic mechanism facilitates it to affect various cells in the body, potentially leading to cognitive impairment even after therapy discontinuation (4).

Chemotherapy can cause cognitive impairments, including memory complaints, reduced attentional processing, clarity of thought, executive functioning issues, and slowed information processing speed (5-9). The rising incidence of BC and prolonged survival attributed to therapeutic advancements underscore the necessity to comprehend the adverse consequences of chemotherapy, especially considering the potential implication of cognitive impairment on the quality of life in this population (10).

Studies have been conducted to evaluate the impact of chemotherapy on cognition in women (7,9). However, the results are scarce and present some inconsistencies, especially regarding treatment duration and evidence of the relationship between side effects and chemotherapy (11,12). This can be explained by the challenges in standardizing and controlling interventions, intervening variables, and understanding the clinical profile, considering intra-individual variability (13-15). Considering that most participants in these studies underwent multiple anticancer treatments during evaluation, the conclusions need to be interpreted with caution (16,17). In addition, the lack of test standardization poses a challenge to the presented evidence.

To gather a thorough comprehensive understanding of the existing evidence in the literature, we conducted a systematic meta-analysis to explore how chemotherapy influences cognitive functions in women with BC. Additionally, a meta-regression analysis was also conducted to assess the covariates that potentially mediate the outcomes of the studies.

## Material and Methods

This is a systematic review with meta-analysis and meta-regression. The study followed the Preferred Reporting Items for Systematic Reviews and Meta-analysis (PRISMA) (18) and the protocol was registered in PROSPERO: Protocol ID: CRD42022301876 (https://www.crd.york.ac.uk/prospero/display_record.php?RecordID=301876). The PICO strategy was used to formulate the research question, as follows: P=Women with breast cancer; I=Chemotherapy treatment; C=No treatment (healthy women); O=Cognitive impairment.

### Search strategy

The systematic search was carried out in the following databases: PubMed, Web of Science, PsycINFO, CINAHL, and Scopus. Articles published between 2012 and May 2022 were selected. The following descriptors were used: ((“cognitive dysfunction” OR “cognitive impairment” OR “cognitive decline”) AND (“breast neoplasm” OR “breast tumor” OR “breast cancer” OR “breast carcinoma” OR “mammary cancer” OR “breast malignant neoplasm” OR “breast malignant tumor” OR “cancer of breast” OR “human mammary carcinoma”) AND (“drug therapy” OR “chemotherapy” OR “chemotherapies” OR “neoadjuvant therapy” OR “neoadjuvant treatment” OR “adjuvant chemotherapy”)). Descriptors according to Medical Subject Headings (MeSH) were used.

### Eligibility criteria

Inclusion criteria were: 1) women with breast cancer; 2) age ≥18 years; 3) received chemotherapy treatment; 4) assessment of cognitive functions through objective and validated tests; 5) manuscripts in English; 6) articles published in the last 10 years (2012-2022), as it is an update review. Exclusion criteria were: 1) animal studies; 2) letters, editorials, literature reviews, systematic reviews, and meta-analyses; 3) behavioral intervention studies; and 4) studies that did not have a control group.

### Procedures

First, two authors (G.S.V.T. and R.G.F.) evaluated titles and abstracts during the first screening and excluded studies that did not meet the eligibility criteria. For each potential study, these two authors evaluated the full texts based on inclusion and exclusion criteria. A third evaluator was contacted (M.E.C.O.) in case of disagreement. Additional searches were performed in the references of all selected articles. Authors were contacted to obtain missing information and any unpublished data.

### Evaluation of the risk of bias

The studies were evaluated according to ACROBAT-NRSI (A Cochrane Risk Of Bias Assessment Tool for Non-Randomized Studies) guidelines (19). The guidelines encompass aspects such as confounding variables, participant selection, measurement of the intervention, non-receipt of the included intervention, losses, measurement of outcomes, and selective reporting of outcomes. Studies are classified as low, medium, or high risk of bias. Studies with at least four high- or low-reported risk domains were excluded from the meta-analysis (Supplementary Figure S1). The evaluation was performed by two independent authors (G.S.V.T. and R.G.F).

### Quality evaluation

The GRADE guidelines were used to assess the evidence quality, which is a widely used strategy in systematic reviews with meta-analysis (20). The factors that influenced the quality of evidence consisted of methodological limitations in design and execution, inconsistency (heterogeneity), indirect evidence, imprecision, and publication bias. An evaluator (O.M.) performed the evaluation of the quality of evidence, and found a moderate level of certainty for the results (Supplementary Table S1).

### Data analysis

A random effects meta-analysis was performed considering the heterogeneity of the tests used for cognitive assessment to calculate the standardized mean difference for the primary outcome (cognitive impairment). Statistical significance was considered when P<0.05. The chi-squared test and the I^2^ statistic were used to assess heterogeneity, in which I^2^ with values above 75% and P<0.05 indicate significant heterogeneity. The random effects model was used to extract the pooled estimates.

Forest plots and the Egger test were used to assess the quality of evidence and the potential for small study effects. The forest plot is a graphical representation of the results of the meta-analysis. The figure summarizes the results in mean and standard deviation for the clinical group and the control group, as well as the standardized mean difference between the two. The diamond symbol in the graph can be used to understand the summarized effect of the analysis, generating indices that help in the interpretation of the graph (21). We used the RevMan (5.4.1) (Cochrane, UK) software program to conduct the meta-analysis.

Meta-regression analysis was performed using the Comprehensive Meta-Analysis (v3) software program (22) in order to analyze the relationship between chemotherapy and cognitive impairment. The sociodemographic and clinical characteristics of the participants were used as covariates in the predictor model, in which: y=cognitive score, x1=age, x2=education, and x3=number of cycles performed. The results are reported as odds ratio (OR) and the 95%CI. A P-value of <0.05 was considered significant.

## Results

**Figure 1:**
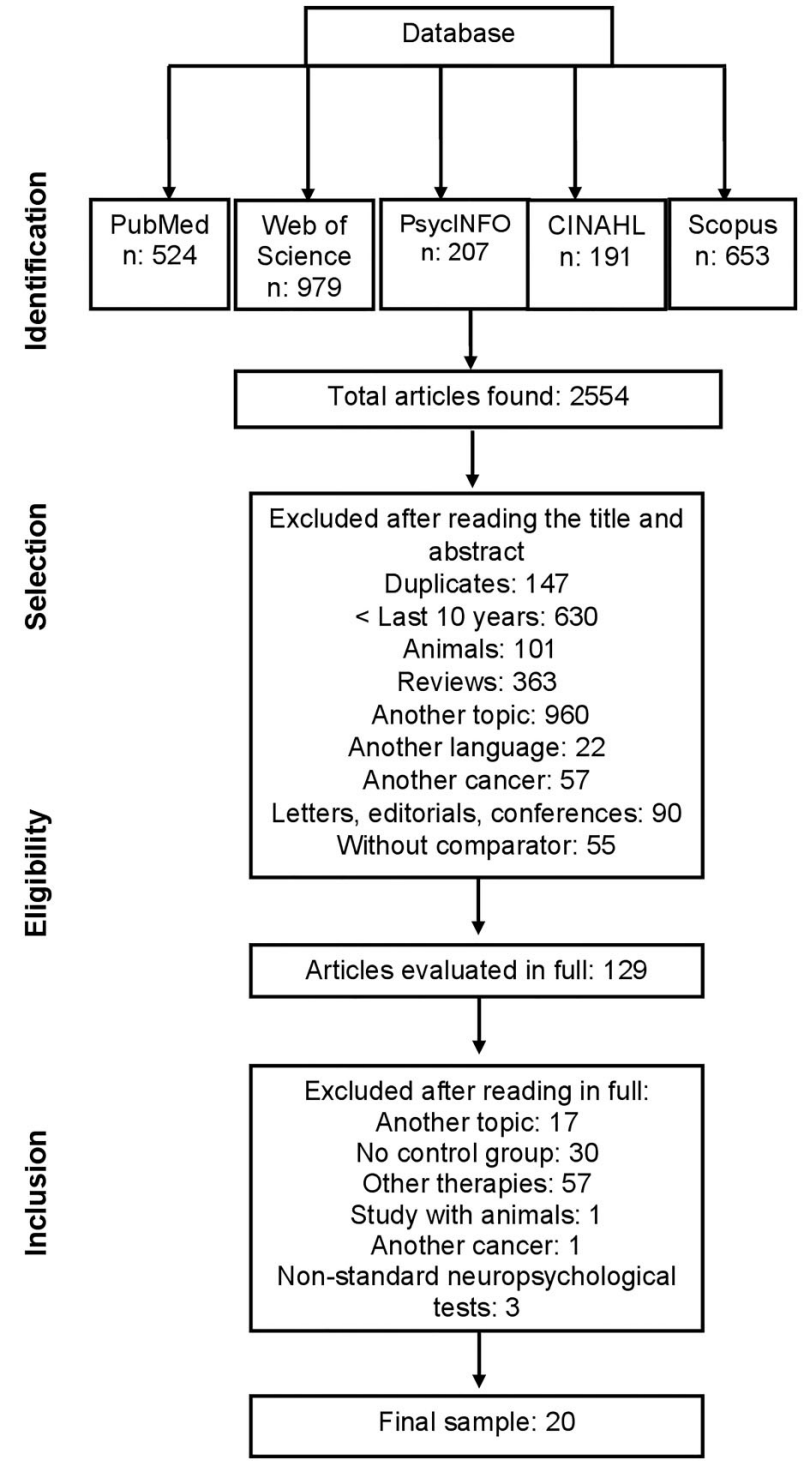
Flowchart of article selection for the systematic review.

The search strategy identified 2,554 articles for analysis in the databases. After screening, 129 articles were selected for full reading. Of these, 109 did not meet the eligibility criteria and were excluded, leaving 20 articles in the final sample. No additional studies were included from the analysis of references (Figure 1).

All included articles were published between 2012 and 2022. Of the studies found, 10 (50%) were conducted in China, 5 (25%) in the United States, 1 (5%) in Ethiopia, 1 (5%) in Poland, 1 (5%) in Canada, 1 (5%) in South Korea, and 1 (5%) in Belgium. The mean age of participants with breast cancer was 49.4±8.2 years, and that of the control group was 48.2±7.6 years. The number of chemotherapy cycles ranged from two to 13. Anxiety and depression scores were assessed in 15 (65%) of the analyzed studies. Information on participants' menopausal status was presented in 10 (50%) of the studies. Supplementary Table S2 presents information about the articles included in this review (6,23-36,38-41,46).

The studies assessed several cognitive domains. The main domains evaluated are presented below, followed by the tests used for their evaluation: i) Inhibitory control - Flanker Inhibitory Control and Attention Test (23); ii) Working memory - List Sorting Working Memory Test, NIH Toolbox for Cognition, WAIS-IV Digit Span (6,23-27); iii) Attention - Trail Making Test A and B, Stroop (6,28-32); iv) Processing speed - Wechsler Adult Intelligence Scale - Fourth Edition (6,24,26); and v) Verbal fluency - Verbal Fluency Test (27,33,34).

The treatment regimens used by the participants varied. Table 1 presents information about the drugs used and the number of women who received each chemotherapy regimen.

**Table 1:** Description of the chemotherapy treatment regimens used by the women evaluated in the studies.

Chemotherapy regimen	N
5-Fluorouracil, epirubicin, cyclophosphamide, taxotere	60
Doxorubicin, cyclophosphamide, paclitaxel	53
Docetaxel, ciclofosfamida, adriamycin	44
Doxorubicin, paclitaxel, cyclophosphamide, 5-fluorouracil	40
Doxorubicin, cyclophosphamide	30
Doxorubicin, paclitaxel	28
Doxorubicin, cyclophosphamide, docetaxel	28
5-Fluorouracil, epirubicin, cyclophosphamide, paclitaxel	19
Epirubicin, cyclophosphamide, paclitaxel	19
Docetaxel, epirubicin, cyclophosphamide	11
Docetaxel, cyclophosphamide	8
Paclitaxel, trastuzumab	4
Carboplatin, paclitaxel	1

**Figure 2:**
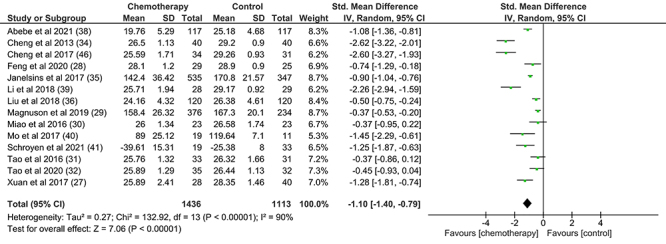
Effect of chemotherapy on the general cognition of women with breast cancer. IV: Random effects; P<0.05: statistically significant difference; I^2^: 0% indicates no heterogeneity across studies, around 25% indicates low heterogeneity, around 50% indicates moderate heterogeneity, and around 75% indicates high heterogeneity across studies.

The first meta-analysis performed in the present study evaluated overall cognitive performance in 1,436 women with breast cancer undergoing chemotherapy and 1,113 healthy controls. Any test that evaluated a cognitive measure was considered for general cognition. Thus, 14 studies were eligible for analysis. The results showed that the chemotherapy group had lower scores for general cognition compared to the control group (-1.10 [95%CI: -1.81 to -0.74], P<0.01). The analysis showed high heterogeneity (χ^2^= 133.82; P<0.01; I^2^: 90%) (Figure 2).

We performed a subgroup analysis with women under the age of 50 years in order to control for the effect of age in the meta-analysis results for general cognition, as there is evidence of cognitive decline related to the aging process (37).

**Figure 3:**
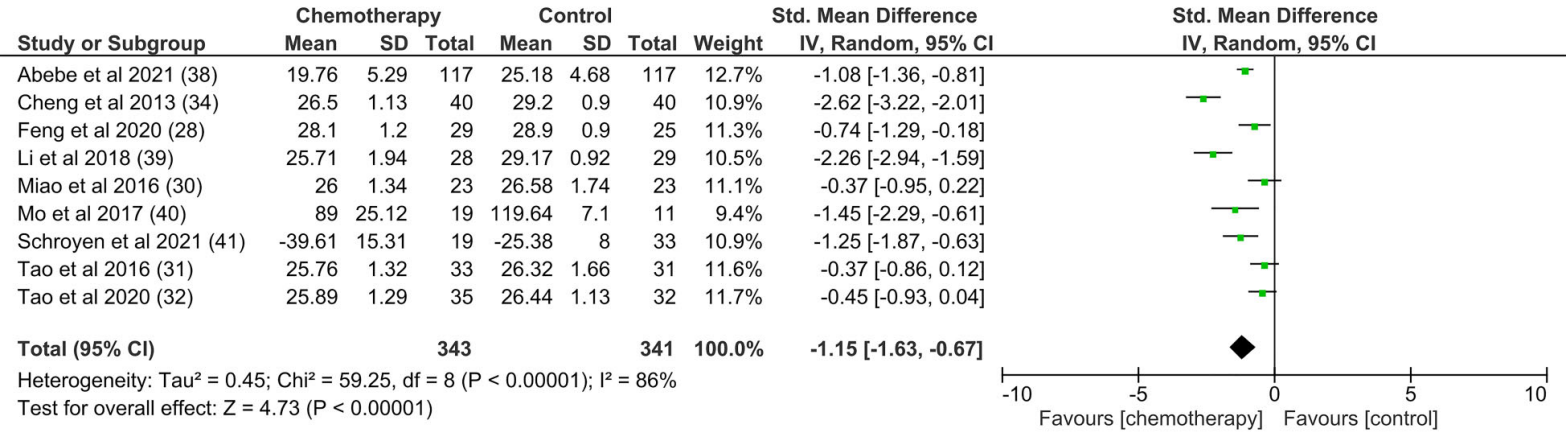
Effects of chemotherapy in women with breast cancer under 50 years of age. IV: Random effects; P<0.05: statistically significant difference; I^2^: 0% indicates no heterogeneity across studies, around 25% indicates low heterogeneity, around 50% indicates moderate heterogeneity, and around 75% indicates high heterogeneity across studies.

The analysis was performed with nine studies composed of women under 50 years of age, totaling 343 women in the clinical group and 341 controls (28,30-32,34,38-41). The results indicated that women younger than 50 years who received chemotherapy performed lower on tests that assessed general cognition compared to the control group (-1.15 [95%CI: -1.63 to -0.67], P<0.01) (Figure 3). This result suggests the influence of chemotherapy on the general cognition of the evaluated women, regardless of age. However, a high heterogeneity among the analyzed studies was found, and these data must be further investigated (χ^2^= 59.25; P<0.01; I^2^: 86%).

**Figure 4:**
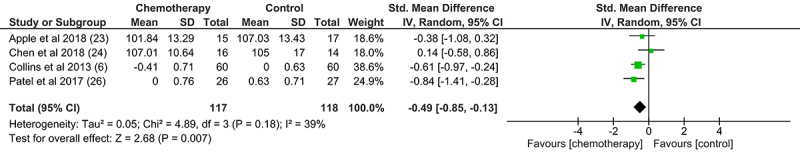
Effects of chemotherapy on working memory in women with breast cancer. IV: Random effects; P<0.05: statistically significant difference; I^2^: 0% indicates no heterogeneity across studies, around 25% indicates low heterogeneity, around 50% indicates moderate heterogeneity, and around 75% indicates high heterogeneity across studies.

We performed a second meta-analysis considering working memory as the outcome. The analysis included four articles, providing a sample of 117 women who received chemotherapy and 118 controls. The results indicated that the clinical group had working memory impairments compared to the control group (-0.49 [95%CI: -0.85 to -0.13], P=0.03). Although the results suggest substantial heterogeneity, it was not statistically significant (χ^2^= 4.50; P=0.11; I^2^: 56%) (Figure 4).

To understand the heterogeneous sources of the included articles and the covariates that interfered with the overall cognition outcome, meta-regressions were performed. The following were considered as covariates: age, education, number of cycles performed, and anxiety and depression scores. Only the education variable showed a relationship with general cognition scores (Q=4.85, df=1, P<0.02), and is a factor that is well established in the literature (42-45). Thus, women with breast cancer who have lower education levels seem to have worse cognitive performance. These data corroborated the findings of a previous study that found an association between lower education level and worse Mini-Mental State Examination (MMSE) performance in women with breast cancer (38).

Although the anxiety and depression variables did not show significance in the meta-regression analysis in the present study, this is a point that deserves attention. Only seven of the studies included in the meta-analysis model for general cognition provided the mean scores for this variable in their studies (28-32,40,41). This was followed by two articles that reported excluding participants who had indices outside the normal range (34,46). Thus, seven studies did not provide data on anxiety and depression for their participants.

## Discussion

Cognitive deficits resulting from chemotherapy have been frequently reported in the literature (9,38,47,48). However, the way in which cognitive domains are affected varies depending on the cytotoxic agent used, which can be explained by differences in the mechanisms of action of the various agents (49).

These results have also been found in studies with animal models. In this regard, a study carried out with mice sought to assess the neurobiological mechanisms underlying the cytotoxic phenomenon of chemotherapy (50). The results suggested that animals treated with docetaxel and doxorubicin regimens had increased blood vessels in the hippocampus and prefrontal cortex after three weeks of treatment. In addition, drug administration with cyclophosphamide, docetaxel, 5-fluorouracil (5-FU), and topotecan triggered a decrease in the number of microglial cells in the prefrontal cortex (50).

5-FU is a chemotherapeutic agent widely used in breast cancer patients (49). It acts as an antimetabolite so as to prevent cell proliferation and partly inhibit the thymidylate synthase enzyme, blocking the formation of thymidine necessary for DNA synthesis (51). Despite having a short half-life of <30 min, it reaches the brain via passive diffusion and its effects can last for months or years (52).

Among the main effects resulting from 5-FU presented in the literature are losses in working memory, executive functions, and attentional aspects (53,54). Biochemical and structural changes in the brain were also presented, such as apoptosis and decreased hippocampal neurogenesis, considered a primary mediator of executive functions (55). In addition, an overall decrease in dopamine release in the striatum, prefrontal cortex, and hippocampus was also shown (54,56,57). The presence of dopamine in the hippocampus is associated with learning, working memory, and long-term memory formation. Thus, changes in this neurotransmitter can influence these cognitive functions (58). Doublecortin levels, considered a modulator of cell survival, were also affected with 5-FU induction (59).

Changes in the working memory pattern have been reported in breast cancer patients undergoing chemotherapy (17,60). These findings are consistent with neuroimaging studies performed on cancer survivors, which identified changes in the dorsal and medial prefrontal cortex and anterior white matter, constituting structures related to working memory and information encoding (61-63).

The biological mechanisms involved in brain changes due to cancer and chemotherapeutic agents used in treating the disease are oxidative stress, DNA damage, and compromised DNA repair (64). In addition, there is evidence of increased oxidative DNA damage to white blood cells in women with breast cancer who have not yet undergone treatment. However, these changes are greater after chemotherapy (65). Direct DNA damage can occur as a result of the mechanism of action of the administered drug, as is the case with doxorubicin, which acts by intercalating with DNA in order to break the strand of nucleotides that carry genetic information (61).

Depression has been linked to neuroplasticity failure, neuronal atrophy, and synaptic decrease in the medial prefrontal cortex, triggering cognitive impairment and prefrontal inhibition (66-68). In turn, pathological anxiety appears to be related to structural degeneration and damage to the hippocampus and prefrontal cortex, which may increase the risk of developing neuropsychiatric disorders (69). The occurrence of anxiety and depression in breast cancer patients is well established in the literature (70), and this seems to be more expressive in patients who have low dopamine and serotonin levels (71). Thus, this condition should be further explored when analyzing the relationship between chemotherapy and cognitive impairment in order to better control this possible mediating effect.

This study had some limitations. The inclusion of studies published in the last ten years restricted the number publications. However, the choice of this period is justified as this scientific report was intended to update the literature on the subject. In addition, the included studies presented several non-standardized data, especially on factors such as anxiety, depression, and menopausal status, which are relevant and can influence the analyzed outcome, and high heterogeneity.

The limited number of controlled studies on chemotherapeutic agents in the treatment of breast cancer makes it difficult to understand the activity of these drugs on cognitive performance. Finally, we were unable to specify the dose-response effects of chemotherapy on cognitive function and on specific domains, as the studies did not provide robust data on the number of sessions that participants received, which is essential for the purpose of the study. However, the presented analyses clarified important aspects of the relationship between chemotherapy and cognitive impairment in women with breast cancer, also showing the mediating variables for the analyzed outcome.

The findings of this meta-analysis confirmed the influence of chemotherapy on the overall cognitive decline of women with breast cancer, especially when they have a low education level. Possible treatments to reverse the cognitive changes arising from antineoplastic treatment have been tested through non-invasive neuromodulation, which has shown promising results (72). In addition, an increasing number of non-pharmacological interventions are being suggested for breast cancer survivors. It is believed that these results may improve survival in women undergoing chemotherapy (73).

Our results draw attention to the impact that this adverse effect can have on the social life and productivity of women, especially younger women, as they are also affected by cognitive impairment and may perform worse in their work activities. Thus, in addition to affecting the performance of daily activities, cognitive changes can also result in economic losses. Despite side effects, chemotherapy is still one of the main antineoplastic treatments, improving survival in women with breast cancer. Knowing the pharmacological profile of substances used, the clinical characteristics of women, along with an interdisciplinary follow-up, can help improve the quality of life after treatment.

Further studies should be conducted in a prospective and detailed manner to better understand the chemotherapy effects on cognitive function of women with breast cancer and improve clinical practice to reveal predictors of cognitive impairment related to chemotherapy in this population.

## Supplementary Material

Click to view [pdf].
